# A comparative study of the impact of lineal relatives versus collateral relatives donor on prognosis in haplo-HSCT for aplastic anemia

**DOI:** 10.3389/fimmu.2026.1727816

**Published:** 2026-04-22

**Authors:** Zhengwei Tan, Ningning Zhu, Yuechao Zhao, Huijin Hu, Qinghong Yu, Yu Zhang, Tonglin Hu, Dijiong Wu, Baodong Ye, Wenbin Liu

**Affiliations:** 1 Department of Hematology, The First Affiliated Hospital of Zhejiang Chinese Medical University (Zhejiang Provincial Hospital of Traditional Chinese Medicine);, Hangzhou, China; 2 The First School of Clinical Medicine, Zhejiang Chinese Medical University;, Hangzhou, China

**Keywords:** aplastic anemia, collateral relatives, donor relationship, haploidentical hematopoietic stem cell transplantation, lineal relatives

## Abstract

**Objective:**

To investigate the impact of Lineal and Collateral donors on the prognosis of recipients in haplo-HSCT for AA among relatives.

**Methods:**

A retrospective analysis of data from AA patients who underwent haplo-HSCT at our center from January 2018 to January 2025. Patients were grouped based on the kinship between donors and recipients (Lineal vs. Collateral), and comparisons were made across key indicators, including engraftment, viral reactivation, GVHD, OS, and GRFS.

**Results:**

The study included 190 AA patients who received haplo-HSCT. Based on donor type, they were divided into the Lineal group (134 patients) and the Collateral group (56 patients). The results showed that the 3-year OS (92.8% vs. 75.3%, *P* = 0.004) and 3-year GRFS (78.5% vs. 64.9%, *P* = 0.062) were relatively higher in the Collateral group compared to the Lineal group. Further analysis revealed that younger donors (age ≤ 40 years) and younger patients (age ≤ 40 years) contributed significantly to improved outcomes in the Collateral group, with both 3-year OS and GRFS being significantly better than in the Lineal group.

**Conclusion:**

The study suggests that Collateral donors represent a viable and effective option, particularly in the absence of Lineal donors. The age of the donor and recipient is a critical factor influencing transplantation outcomes, with younger donors and patients potentially enhancing therapeutic efficacy.

## Introduction

Aplastic anemia (AA) is a syndrome of bone marrow failure caused by multiple etiologies, characterized clinically by marrow hypoplasia, pancytopenia, and symptoms such as anemia, bleeding, and infection (1, 2). Based on disease severity, AA is classified into non-severe (NSAA), severe (SAA), and very severe (VSAA). SAA and VSAA are life-threatening conditions associated with poor prognosis in the absence of timely and effective intervention. For younger patients with a human leukocyte antigen (HLA)-matched sibling donor (MSD), allogeneic hematopoietic stem cell transplantation (allo-HSCT) is the first-line treatment (3). However, only approximately 25%~30% of patients can find an MSD, leaving most unable to receive first-line transplantation due to a lack of suitable donors (4). For patients without an MSD, anti-thymocyte globulin (ATG) combined with cyclosporine (CsA)-based immunosuppressive therapy (IST) represents the established first-line treatment approach. Nevertheless, IST has limitations in efficacy, with some patients experiencing treatment failure, relapse, or late clonal evolution (5, 6). Despite advances in treatment, identifying effective alternative therapies for SAA patients lacking an MSD remains a major challenge in this field.

With continuous advancements in transplantation techniques, haploidentical hematopoietic stem cell transplantation (haplo-HSCT) has emerged as an effective solution to the donor shortage problem, offering new hope for SAA patients without an MSD. Haploidentical donors are usually relatives sharing half of the HLA genes, such as parents, children, or siblings, which significantly expands the donor pool. Multiple studies have shown that the overall survival (OS) and failure-free survival (FFS) rates of haplo-HSCT for SAA are comparable to those of MSD-HSCT (7, 8). Consequently, haplo-HSCT not only plays a crucial role in salvage therapy after IST failure but is also increasingly recommended as a first-line treatment for patients without an MSD, potentially transforming the traditional treatment landscape.

Despite the maturation of haplo-HSCT technology, donor selection remains a critical factor influencing transplant success and long-term survival (9, 10). When multiple potential haploidentical donors are available, determining the optimal donor to minimize transplant-related complications and improve quality of life is an urgent issue in clinical practice. Current research on donor selection primarily focuses on malignant hematological diseases, with relatively limited and inconsistent studies in non-malignant diseases like SAA. Some studies suggest that paternal donors may be associated with lower GVHD risk and better survival outcomes compared to maternal donors, possibly due to maternal micro-chimerism and immune tolerance (11). Similarly, comparisons between child donors and sibling donors have shown inconsistent results, with some studies indicating potential advantages of child donors (12). Therefore, clarifying the specific impacts of different relatives on SAA patient outcomes is crucial for developing refined donor selection strategies. Based on this background, we conducted a retrospective clinical analysis to systematically compare the clinical outcomes of SAA patients undergoing haplo-HSCT with Lineal (LDs) versus Collateral donors (CDs). The aim was to provide higher-level evidence-based medical evidence for clinical donor selection, particularly for Collateral donors, offering valuable guidance for clinical practice.

## Patients and methods

### Patients

This retrospective study encompassed 190 AA patients who underwent haplo-HSCT between Jan 2018 and Jan 2025. Among them, 134 donors were LDs (including parents, children, etc.), and 56 donors were CDs (including sibling, etc.). The diagnosis and classification of AA adhered to the Camitta criteria (13). The study was conducted in accordance with the Helsinki Declaration and obtained approval from the Ethics Committee of the First Affiliated Hospital of Zhejiang Chinese Medical University (Approval number: 2025-KLS-845-02). Prior to treatment, all patients and donors provided written informed consent.

Inclusion criteria: 1. Diagnosed with AA, including SAA and VSAA types. 2. Underwent first-time haplo-HSCT. 3. The donor is a relative with haploidentical HLA matching. 4. Complete clinical and follow-up data are available.

### Conditioning regimens

Patients received one of three conditioning regimens: FCA (fludarabine, cyclophosphamide, and ATG), Bucy (fludarabine, cyclophosphamide, busulfan and ATG), or FABT (fludarabine, ATG, busulfan, and thiotepa) (5, 14). The primary difference between groups was that the FABT group received high-dose cyclophosphamide for GVHD prophylaxis, whereas the FCA and Bucy groups received methotrexate. Mesenchymal stem cells (MSCs) and/or umbilical cord blood stem cells (UCBs) were administered as an adjunctive infusion on day −1, followed by bone marrow stem cells.

### Definition and assessment

All outcomes were defined from the time of the first HSCT. The primary endpoints were GVHD-free and relapse-free survival (GRFS) and overall survival (OS). Secondary endpoints included efficacy, engraftment, incidence of GVHD, and viral reactivation. Neutrophil (NE) Engraftment: By day 28 post-transplantation, an absolute NE count reaching 0.5×10^9^/L on three consecutive days. Platelet (PLT) Engraftment: By day 28 post-transplantation, a PLT count maintained above 20×10^9^/L for seven consecutive days without transfusion support. Acute GVHD (aGVHD) and Chronic GVHD (cGVHD): Classified and graded according to the internationally recognized Glucksberg-Seattle criteria (15). CMV Reactivation: CMV-DNA copy number in plasma sample exceeding 1,000 copies/mL on two consecutive occasions. EBV Reactivation: EBV-DNA copy number in whole blood samples exceeding 10,000 copies/mL on two consecutive occasions. GRFS was defined as survival without disease relapse, grade III-IV aGVHD, or extensive cGVHD requiring systemic treatment (16). OS: the time from HSCT to death or last follow-up.

Efficacy grading according to NIH criteria (17). Complete response (CR): HGB > 100 g/l, PLT > 100 × 10^9^/l, ANC > 1.5 × 10^9^/l, not dependent on blood transfusion; Partial response (PR): withdrawal from component blood transfusion (HGB > 70 g/l, PLT > 20 × 10^9^/l), improvement of hematological indices, no longer fulfilling SAA diagnostic criteria; No response (NR): Patients who were continuously transfusion-dependent and/or still fulfilled the diagnostic criteria for SAA and who died from various causes after HSCT; Overall response (OR):CR + PR.

### Statistical analysis

The data analysis was conducted using SPSS version 27.0 (IBM, Armonk, NY) and Prism version 8.0 (GraphPad Software, San Diego, CA). Continuous variables that did not adhere to a normal distribution were reported as the median (range) and were compared utilizing the rank-sum test. Categorical variables were presented as frequencies and compared using the chi-square test. Specifically: For OS and GRFS, the Kaplan-Meier method was used to estimate survival probabilities, and comparisons between groups were performed using the log-rank test. The cumulative incidences of engraftment, viral reactivation, and GVHD were analyzed using cumulative incidence functions, treating death without the event as a competing risk, and compared between groups using Gray’s test. For univariate and multivariate analyses, Cox proportional hazards regression models were used for OS and GRFS. For aGVHD and cGVHD, Fine-Gray subdistribution hazard models were applied to account for competing risks (death without the event). Variables with a *P*-value < 0.10 in univariate analysis or those considered clinically relevant were included in the multivariate models. Two-tailed *P*-values were used for all statistical comparisons. A *P*-value of less than 0.05 was considered to indicate statistical significance.

## Results

### Patient characteristics

A total of 190 AA patients who underwent haplo-HSCT were enrolled in this study. Based on the genetic relationship between donors and recipients, patients were divided into the LDs group (134 cases) and the CDs group (56 cases). Between the two groups, there were statistically significant differences in the age distribution of donors and recipients. However, no statistically significant differences were observed in other aspects, such as gender ratio, disease stratification, blood type compatibility, or conditioning regimens. The detailed baseline data of the patients in the two groups are shown in **Table 1**.

**Table 1:** Baseline data of two groups.

Characteristic	Lineal group	Collateral group	*P* value
Number	134	56	
Sex, n (%)			0.433
Male	73 (54.5)	27 (48.2)	
Female	61 (45.5)	29 (51.8)	
Age, yr, median (range)	31 (8~74)	31 (10~49)	**0.001**
Donor sex, n (%)			0.135
Male	90 (67.2)	31 (55.4)	
Female	44 (32.8)	25 (44.6)	
Donor, Age, yr, median (range)	28 (5~55)	27 (10~47)	**0.007**
Blood values before HSCT, median (range)			
WBC	1.7 (0~3.4)	1.7 (0.1~3.4)	0.153
ANC	0.4 (0~1.4)	0.4 (0~2.1)	0.186
HGB	57 (14~77)	57 (29~71)	0.676
PLT	12 (1~38)	11 (1~44)	0.496
Ferritin	791 (65~17747)	779 (37~5866)	0.062
Disease classification, n (%)			0.912
SAA	73 (54.5)	31 (55.4)	
VSAA	61 (45.5)	25 (44.6)	
Interval from diagnosis to HSCT, n (%)			0.431
≤1yr	94 (70.1)	36 (64.3)	
>1yr	40 (29.9)	20 (35.7)	
HCT-CI, n (%)			0.064
0	87 (64.9)	45 (70.4)	
≥1	47 (35.1)	11 (29.6)	
ABO mismatch, n (%)			0.576
Match	73 (54.5)	33 (58.9)	
Mismatch	61 (45.5)	23 (41.1)	
HLA, n (%)			0.913
5/12	21 (15.7)	16 (28.6)	
6/12	62 (46.3)	18 (32.1)	
7/12	40 (29.9)	11 (19.6)	
8/12	9 (6.7)	9 (16.1)	
9/12	2 (1.4)	2 (3.6)	
PNH, n (%)	9 (6.7)	6 (10.7)	0.354
Donor/Recipient EBV serology			0.096
+/+	131 (97.7)	56 (100)	
-/+	3 (2.3)	NA	
Donor/Recipient CMV serology			0.322
+/+	134 (100)	55 (98.2)	
-/+	NA	1 (1.8)	
Previous IST treatment, n (%)	8 (5.9)	3 (5.4)	0.607
Graft source, n (%)			0.464
PBSCs	8 (6.0)	5 (8.9)	
BMSCs + PBSCs	126 (94.0)	51 (91.1)	
Conditioning regimen, n (%)			0.862
FABT	36 (26.9)	13 (23.2)	
FCA	74 (55.2)	34 (60.7)	
Bucy	24 (17.9)	9 (16.1)	
UCBs, n (%)	54 (40.3)	20 (35.7)	0.557
MSCs, n (%)	60 (44.8)	29 (51.8)	0.380
CD34+ cell count, ×10^6^/kg, median (range)	6.0 (1.69~20.01)	6.0 (2.89~25.38)	0.142
Letermovir, n (%)	32 (23.9)	13 (23.2)	0.922

Significant *P* values are in bold type.

WBC, White Blood Cell; ANC, Absolute Neutrophil Count; HGB, Hemoglobin; PLT, Platelet; PBSCs, Peripheral Blood Stem Cell; BMSCs, Bone Marrow Stem Cell; DSA, Donor Specific Antibody; PNH, Paroxysmal Nocturnal Hemoglobinuria; NA, Not Available.

### Transplantation outcomes

**Figure 1:**
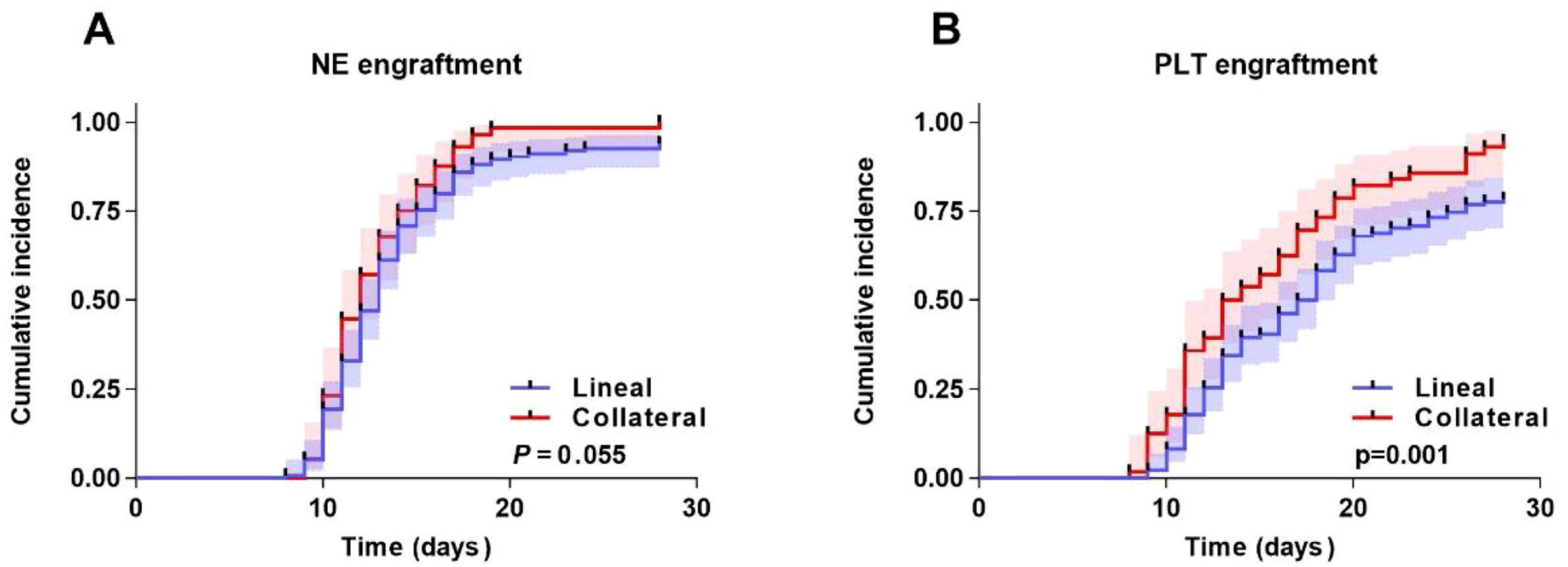
Transplantation outcomes after haplo-HSCT in two groups. **(A)** NE engraftment. **(B)** PLT engraftment. Shaded areas represent 95% confidence intervals.

The median number of CD34+ cells in the grafts was 6.0×10^6^/kg (range: 1.69~20.01) for the Lineal group and 6.0×10^6^/kg (range: 2.89~25.38) for the Collateral group, with no significant difference between the two groups (*P* = 0.142). The cumulative NE engraftment rate within 28 days was 94.1% (95%CI, 88.6%~99.7%) in the Lineal group and 100.0% (95%CI, 91.7%~100%) in the Collateral group (*P* = 0.055) (**Figure 1A**). The cumulative PLT engraftment rate within 28 days was 78.4% (95%CI, 70.6%~89.2%) in the Lineal group and 94.6% (95%CI, 88.9%~99.7%) in the Collateral group (*P* = 0.001) (**Figure 1B**).

**Figure 2:**
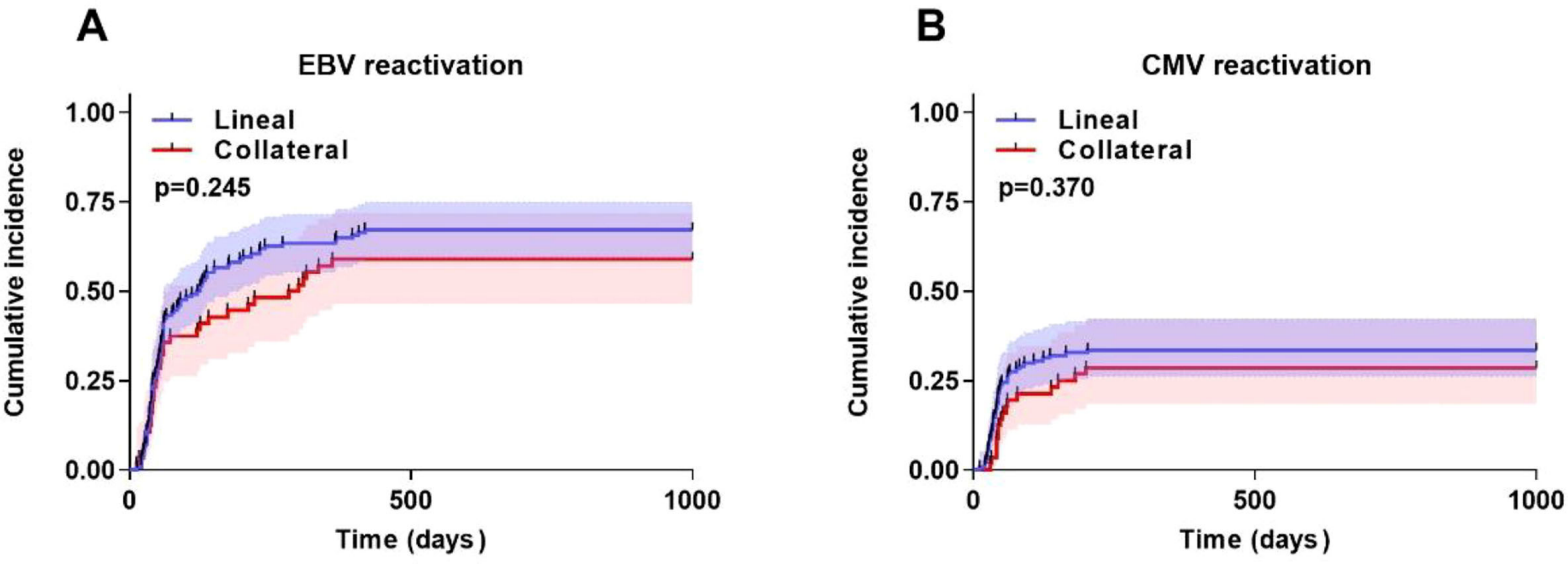
Transplantation outcomes after haplo-HSCT in two groups. **(A)** EBV-reactivation. **(B)** CMV-reactivation. Shaded areas represent 95% confidence intervals.

The cumulative EBV reactivation rate was 67.1% (95%CI, 51.1%~75.6%) in the Lineal group and 58.9% (95%CI, 43.5%~69.5%) in the Collateral group (*P* = 0.245) (**Figure 2A**). Similarly, the cumulative CMV reactivation rate was 33.5% (95%CI, 18.7%~45.1%) in the Lineal group and 28.5% (95%CI, 13.5%~41.5%) in the Collateral group (*P* = 0.370) (**Figure 2B**). However, no patients in either group progressed to CMV disease.

**Figure 3:**
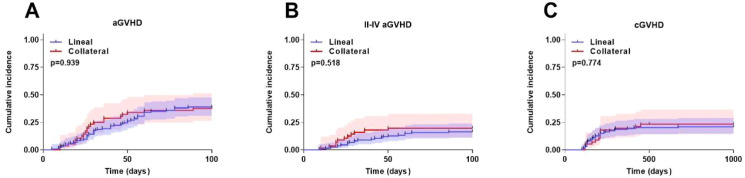
Transplantation outcomes after haplo-HSCT in two groups. **(A)** aGVHD. **(B)** II-IV aGVHD. **(C)** cGVHD. Shaded areas represent 95% confidence intervals.

The cumulative aGVHD rate was 38.3% (95%CI, 21.7%~56.3%) in the Lineal group and 37.5% (95%CI, 21.1%~51.7%) in the Collateral group (*P* = 0.939) (**Figure 3A**). The cumulative II-IV aGVHD rate was 16.4% (95%CI, 13.1%~33.6%) in the Lineal group and 19.6% (95%CI, 7.5%~22.1%) in the Collateral group (*P* = 0.518) (**Figure 3B**). The cumulative cGVHD rate was 20.8% (95%CI, 11.1%~31.9%) in the Lineal group and 23.2% (95%CI, 9.7%~30.5%) in the Collateral group (*P* = 0.774) (**Figure 3C**).

**Figure 4:**
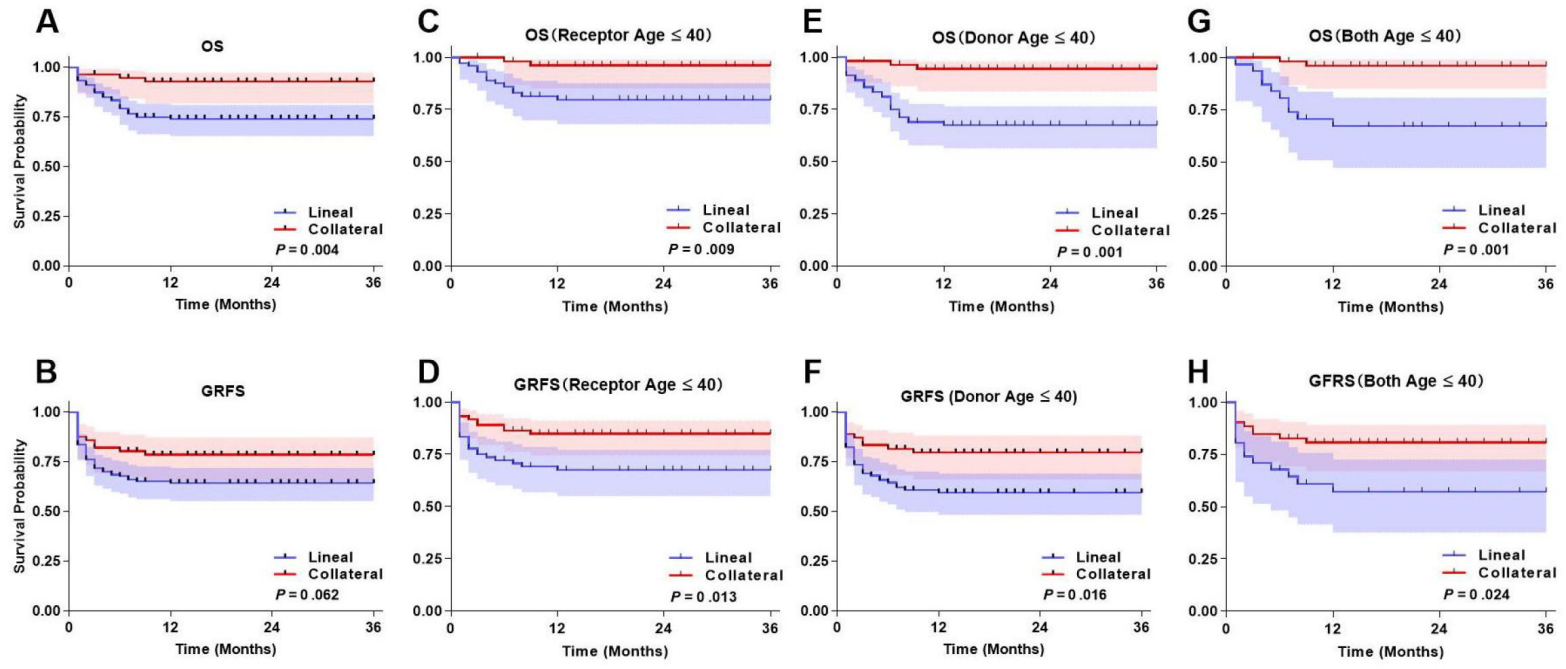
Transplantation outcomes after haplo-HSCT in two groups. **(A)** OS. **(B)** GRFS. **(C)** OS in Receptor age ≤40 group. **(D)** GRFS in Receptor age ≤40 group. **(E)** OS in Donor age ≤40 group. **(F)** GRFS in Donor age ≤40 group. **(G)** OS in Both donor and receptor age ≤40 group. **(H)** GRFS in Both donor and receptor age ≤40 group. Shaded areas represent 95% confidence intervals.

The 3-year OS was 75.3% (95%CI, 64.6%~86.7%) in the Lineal group compared to 92.8% (95%CI, 83.6%~99.7%) in the Collateral group (*P* = 0.004) (**Figure 4A**). The 3-year GRFS was 64.9% (95%CI, 51.3%~73.9%) in the Lineal group compared to 78.5% (95%CI, 65.9%~87.3%) in the Collateral group (*P* = 0.062) (**Figure 4B**).

Due to the significant differences in the age distribution of donors and recipients between the two groups, we reclassified donors and recipients under 40 years of age to compare their OS and GRFS. The results showed that in patients under 40 years of age, the 3-year OS in the Lineal group and Collateral group was 80.5% (95%CI, 67.6%~89.7%) and 96.1% (95%CI, 87.6%~99.7%), respectively (*P* = 0.009) (**Figure 4C**). The 3-year GRFS in the Lineal group and Collateral group was 68.0% (95%CI, 57.6%~78.7%) and 80.7% (95%CI, 67.6%~91.7%), respectively (*P* = 0.013) (**Figure 4D**).

In patients with donors under 40 years of age, the 3-year OS in the Lineal group and Collateral group was 69.2% (95%CI, 57.6%~78.7%) and 94.4% (95%CI, 89.6%~99.9%), respectively (*P* = 0.001) (**Figure 4E**). The 3-year GRFS in the Lineal group and Collateral group was 60.4% (95%CI, 47.8%~71.1%) and 79.6% (95%CI, 65.3%~91.9%), respectively (*P* = 0.016) (**Figure 4F**).

In patients with both donors and recipients under 40 years of age, the 3-year OS in the Lineal group and Collateral group was 67.8% (95%CI, 61.6%~83.7%) and 96.1% (95%CI,89.9%~99.9%), respectively (*P* = 0.001) (**Figure 4G**). The 3-year GRFS in the Lineal group and Collateral group was 58.0% (95%CI, 41.1%~69.7%) and 80.7% (95%CI, 70.3%~92.5%), respectively (*P* = 0.024) (**Figure 4H**).

**Figure 5:**
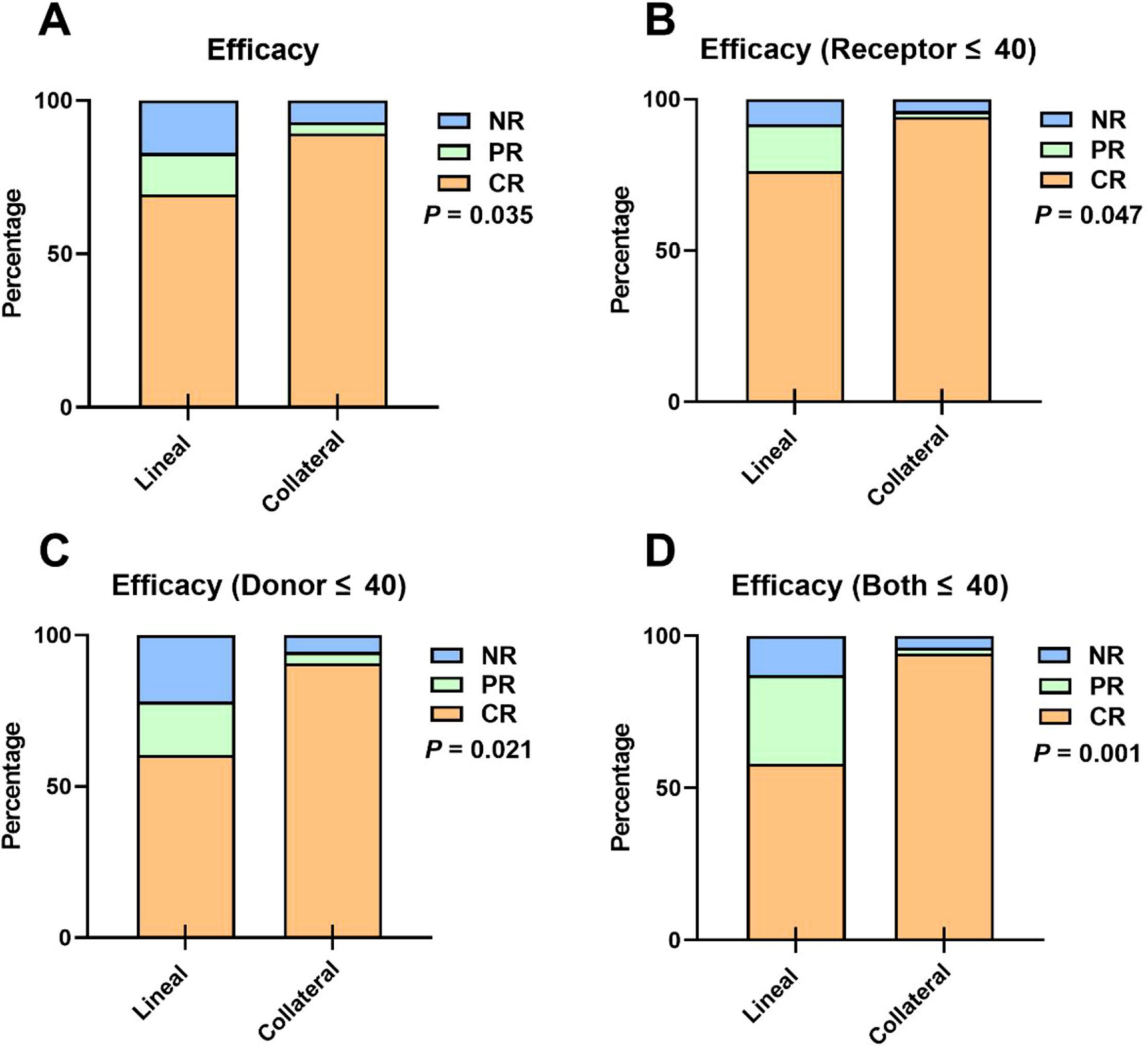
Transplantation outcomes after haplo-HSCT in two groups. **(A)** Efficacy. **(B)** Efficacy in Receptor age ≤40 group. **(C)** Efficacy in Donor age ≤40 group. **(D)** Efficacy in Both donor and receptor age ≤40 group. *P*-values represent global chi-square test comparisons across all response categories (CR, PR, NR) between lineal and collateral donor groups.

In terms of efficacy, the Lineal group exhibited a significantly higher one-year CR rate compared to the Collateral group (69.4% vs. 89.2%) (*P* = 0.035) (**Figure 5A**). Among patients with donors under 40 years of age, the one-year CR rates for the Lineal group and Collateral group were 60.4% and 90.7%, respectively (*P* = 0.047) (**Figure 5B**). In patients under 40 years of age, the one-year CR rates for the Lineal group and Collateral group were 76.3% and 94.2%, respectively (*P* = 0.021) (**Figure 5C**). Among patients with both donors and recipients under 40 years of age, the one-year CR rates for the Lineal group and Collateral group were 58.1% and 94.2%, respectively (*P* = 0.001) (**Figure 5D**).

### Univariate and multivariate analysis

To identify independent predictors of clinical outcomes, we performed multivariate analysis based on clinical relevance and univariate analysis results (variables with *P* < 0.10 in univariate analysis were considered for inclusion) (**Table 2**). For response outcomes, logistic regression was used. For OS and GRFS, Cox proportional hazards models were applied. For aGVHD and cGVHD, Fine-Gray subdistribution hazard models were used to account for competing risks. After adjusting for donor age, recipient age, HCT-CI, CD34+ cell dose, and other baseline covariates, CDs remained a significant favorable factor for Response (OR 0.42, 95%CI 0.21~0.84; *P* = 0.014), OS (HR 0.41, 95%CI 0.22~0.76; *P* = 0.004), and GRFS (HR 0.52, 95%CI 0.30~0.90; *P* = 0.020) compared with LDs.

**Table 2:** Multivariate analysis of the factors associated with Response, II-IV aGVHD, cGVHD, OS, GRFS.

Outcome	Odds ratio/hazard ratio (95%CI)	*P*
Response (OR vs NR)	OR	
Donor Type (Collateral vs Lineal)	0.42 (0.21~0.84)	**0.014**
Donor Age (>40 vs ≤40)	1.89 (1.12~3.19)	**0.017**
Recipient Age (>40 vs ≤40)	2.03 (1.21~3.41)	**0.007**
HCT-CI (≥1 vs 0)	1.76 (1.04~2.98)	**0.036**
CD34+ (>5 vs ≤5 ×10^6^/kg)	0.61 (0.35~1.06)	0.080
ABO mismatch (yes vs no)	1.32 (0.79~2.20)	0.292
UCB infusion (yes vs no)	0.82 (0.49~1.37)	0.445
MSC infusion (yes vs no)	0.78 (0.46~1.32)	0.357
aGVHD (II–IV)	HR	
Donor Type (Collateral vs Lineal)	1.12 (0.58~2.16)	0.735
Donor Age (>40 vs ≤40)	1.43 (0.81~2.52)	0.218
Recipient Age (>40 vs ≤40)	1.27 (0.72~2.24)	0.410
ABO mismatch (yes vs no)	1.58 (0.91~2.74)	0.104
MSC infusion (yes vs no)	0.74 (0.42~1.31)	0.302
UCB infusion (yes vs no)	0.88 (0.57~1.55)	0.664
GVHD Prophylaxis (PTCy vs MTX)	0.78 (0.45~1.35)	0.382
cGVHD	HR	
Donor Type (Collateral vs Lineal)	1.09 (0.53~2.24)	0.817
Donor Age (>40 vs ≤40)	1.38 (0.75~2.54)	0.302
Recipient Age (>40 vs ≤40)	1.19 (0.64~2.21)	0.579
UCB infusion (yes vs no)	0.68 (0.36~1.28)	0.231
MSC infusion (yes vs no)	0.61(0.32~1.11)	0.212
GVHD Prophylaxis (PTCy vs MTX)	0.71 (0.38~1.15)	0.312
OS	HR	
Donor Type (Collateral vs Lineal)	0.41 (0.22~0.76)	**0.004**
Donor Age (>40 vs ≤40)	2.14 (1.25~3.66)	**0.005**
Donor sex (female vs male)	1.19 (0.69~2.05)	0.531
Recipient Age (>40 vs ≤40)	2.31 (1.34~3.98)	**0.002**
HCT-CI (≥1 vs 0)	1.92 (1.12~3.29)	**0.018**
CD34+ (>5 vs ≤5 ×10^6^/kg)	0.59 (0.34~1.02)	0.058
MSC infusion (yes vs no)	0.76 (0.48~1.36)	0.323
UCB infusion (yes vs no)	0.80 (0.46~1.39)	0.428
Conditioning regimen (FABT vs Others)	0.36 (0.12~0.59)	**0.018**
GRFS	HR	
Donor Type (Collateral vs Lineal)	0.52 (0.30~0.90)	**0.020**
Donor Age (>40 vs ≤40)	1.78 (1.08~2.93)	**0.024**
Recipient Age (>40 vs ≤40)	1.96 (1.18~3.26)	**0.009**
HCT-CI (≥1 vs 0)	1.68 (1.02~2.77)	**0.042**
CD34+ (>5 vs ≤5 ×10^6^/kg)	0.64 (0.39~1.05)	0.078
MSC infusion (yes vs no)	0.51 (0.39~1.21)	0.123
UCB infusion (yes vs no)	0.64 (0.41~1.31)	0.328
Conditioning regimen (FABT vs Others)	0.42 (0.21~0.83)	**0.028**

Significant P values are in bold type.

## Discussion

Donor selection is one of the key factors for successful haplo-HSCT in AA patients, particularly since the degree of HLA matching between the donor and recipient directly affects the success of transplantation (18, 19). Recent research has provided deeper insights into the association between donor relationship and transplant prognosis. A 2019 retrospective study by Xu et al. found that four types of LDs, fathers, mothers, siblings, and children, did not significantly impact the prognosis of AA patients (10). However, when patients lack an HLA-identical sibling donor or a suitable LD, CDs become an important alternative option. Previous studies have predominantly focused on hematological malignancies, with limited and inconsistent conclusions regarding non-malignant diseases like AA (11). This study retrospectively analyzed 190 AA patients who underwent haplo-HSCT to systematically compare the impact of lineal versus collateral relative donors on transplant outcomes. The most salient finding was the superior long-term survival in the Collateral group. The 3-year OS for the Collateral group was 92.8%, significantly higher than the 75.3% observed in the Lineal group (*P* = 0.004). A similar trend was noted for 3-year GRFS (78.5% vs. 64.9%, *P* = 0.062). These results indicate that the type of donor relationship in haplo-HSCT may be a significant factor influencing long-term survival.

Further analysis identified donor and recipient age as key prognostic factors. In the subgroup with donors aged ≤40 years, the Collateral group demonstrated significantly superior 3-year OS and GRFS compared to the Lineal group (**Figures 4C, D**). Similarly, significant survival advantages for the Collateral group were observed in subgroups where the recipient was ≤40 years old or both donor and recipient were ≤40 years old (**Figures 4E–H**). This suggests that younger donors may possess enhanced immune reconstitution capacity and lower transplantation-related toxicity, while younger recipients may better tolerate the transplant procedure. This finding aligns with previous emphasis on donor age importance and further supports prioritizing age in donor selection (20, 21). Previous studies have indicated that younger donors may have HSCs with greater proliferative capacity and more robust immune function (22–24), though direct comparisons between lineal and collateral donors of the same age group require further investigation. To further validate the independent role of donor type and adjust for potential confounders, we performed multivariate analyses. After controlling for factors including donor and recipient age, CDs remained significantly associated with superior overall response (OR 0.42, *P* = 0.014), OS (HR 0.41, *P* = 0.004), and GRFS (HR 0.52, *P* = 0.020) compared with LDs. These findings confirm that the use of CDs is an independent protective factor for long-term outcomes in haplo-HSCT for AA.

Regarding engraftment, the cumulative 28-day PLT engraftment rate was significantly higher in the Collateral group (94.6% vs. 78.4%, *P* = 0.001). This suggests that CDs may be more conducive to hematopoietic recovery, particularly PLT reconstitution, possibly related to the discussed differences in intrinsic stem cell quality and bone marrow microenvironment support, jointly determined by donor type and age. Notably, the incidence of GVHD and viral reactivation did not differ significantly between the two groups, indicating that CDs did not increase the risk of immune-related complications. Natural killer (NK) cells play a critical role in early immune reconstitution and anti-infection (25). Their activity is regulated by the interaction between killer-cell immunoglobulin-like receptors (KIR) on NK cells and their corresponding HLA class I ligands on target cells. In haplo-HSCT, a mismatch between donor KIR and recipient HLA ligands may release NK cells from inhibitory signals, thereby enhancing their ability to eliminate residual malignant cells or viruses without increasing the risk of GVHD (26, 27). Due to genetic inheritance patterns, the KIR-ligand mismatch in related donor-recipient pairs is fixed and unidirectional in the case of parent–child or sibling donors with identical haplotypes. In contrast, collateral relatives may exhibit more diverse and advantageous KIR–ligand mismatch profiles as a result of HLA recombination. This diversity could potentially lead to more potent and beneficial immune responses mediated by NK cells from collateral relatives (28). Furthermore, collateral donors might carry specific gene polymorphisms related to immune regulation (e.g., cytokine genes, Toll-like receptor genes), which could influence the intensity of post-transplant inflammatory responses and consequently impact GVHD development (29).

Among LDs, maternal donors share a distinct history of immune tolerance with the recipient due to bidirectional fetal-maternal cellular trafficking during pregnancy (30, 31). This pre-existing immune tolerance may attenuate the graft-versus-host reaction and compromise the clearance of pathogenic T-cell clones critical for AA pathogenesis. However, paternal donors and child donors, while lacking this pregnancy-induced micro-chimerism, may still exhibit distinct biological characteristics compared to CDs. Paternal donors could potentially share partial HLA haplotypes and environmental exposures with the recipient, whereas child donors represent a generational shift that may influence telomere length and stem cell functionality (32). In contrast, unrelated or haploidentical donors generally lack this profound pregnancy-induced immunologic exchange. Their immune recognition is more naive and straightforward, potentially facilitating the establishment of a *de novo* and fully competent immune system in the recipient. This may lead to more thorough clearance of pathogenic immune effectors and improved eradication of the autoimmune processes driving AA. Therefore, while non-maternal lineal donors lack maternal micro-chimerism, they still differ fundamentally from collateral donors in terms of genetic inheritance patterns and KIR-ligand mismatch profiles.

In summary, CDs may contribute to improved hematopoietic recovery and long-term survival without increasing GVHD risk, potentially due to a combination of their distinct KIR-ligand mismatch profiles arising from HLA recombination during meiosis, less pre-established immune tolerance (particularly when compared to maternal donors with prior fetal-maternal micro-chimerism), and generally more youthful cellular biological status compared to LDs. Our results indicate that when multiple potential donors are available, CDs should not be readily excluded, as they can provide long-term survival benefits comparable to, or potentially superior to LDs. In the absence of an HLA-identical sibling donor, CDs are not only a feasible alternative but may in certain scenarios, particularly when both donor and recipient are young, yield superior outcomes. However, this study has limitations. Firstly, it is a single-center, retrospective analysis with a limited sample size, especially in the CDs group, raising the possibility of selection bias. Secondly, the proposed mechanisms (e.g., KIR typing, micro-chimerism levels) were not directly tested. Future prospective studies incorporating biological experiments are warranted to validate these hypotheses.

## Data Availability

The raw data supporting the conclusions of this article will be made available by the authors, without undue reservation.
